# Environmental Stability of Alkyl Phosphate- and Sulfate-Containing Membranes as Early Cell Models

**DOI:** 10.1021/acsearthspacechem.6c00171

**Published:** 2026-09-24

**Authors:** Christelle Ekosso, Hao Liu, Avery Glagovich, Adam Rogers, Lila N. Strober, Joshua Schrier, Sarah Maurer

**Affiliations:** † Department of Chemistry and Biochemistry, 5745 Central Connecticut State University, 1615 Stanley Street, New Britain, Connecticut 06050, United States; ‡ Department of Chemistry and Biochemistry, Fordham University, 441 East Fordham Road, The Bronx, New York 10458, United States; § Department of Chemistry, 1810 Tufts University, 419 Boston Ave, Medford, Massachusetts 02155, United States

**Keywords:** protocells, early Earth, abiogenesis, vesicles, liposomes, permeability

## Abstract

The ability of simple amphiphiles to assemble into stable membranes is central to understanding how protocells may have formed on early Earth. In this work, we examine how small structural differences, specifically hydrocarbon chain length and headgroup identity, influence membrane formation, stability, and permeability in mixed amphiphile systems exposed to diverse conditions to understand the role of the environment in protocell evolution. Using automated sample preparation and confocal microscopy, we characterized eight compositions containing decyl sulfate (DS), dodecyl sulfate (DDS), or dodecyl phosphate (DDP) across a range of pH values in ultrapure water, sea salt, or sea salt mixed with a complex prebiotic organic solution. Longer-chain and phosphate-containing amphiphiles formed more vesicles, particularly at low pH and high ionic strength. DS exhibited consistently low and environment-insensitive critical vesicle concentrations (CVCs), whereas DDP displayed the highest sensitivity to alkaline conditions. DDP-containing vesicles were the least permeable, highlighting the lipid packing influence of the phosphate headgroup. Together, these findings show that small modifications in amphiphile composition strongly affect membrane properties across early Earth-like environments, demonstrating the role of anionic surfactants in protocell emergence.

## Graphical abstract

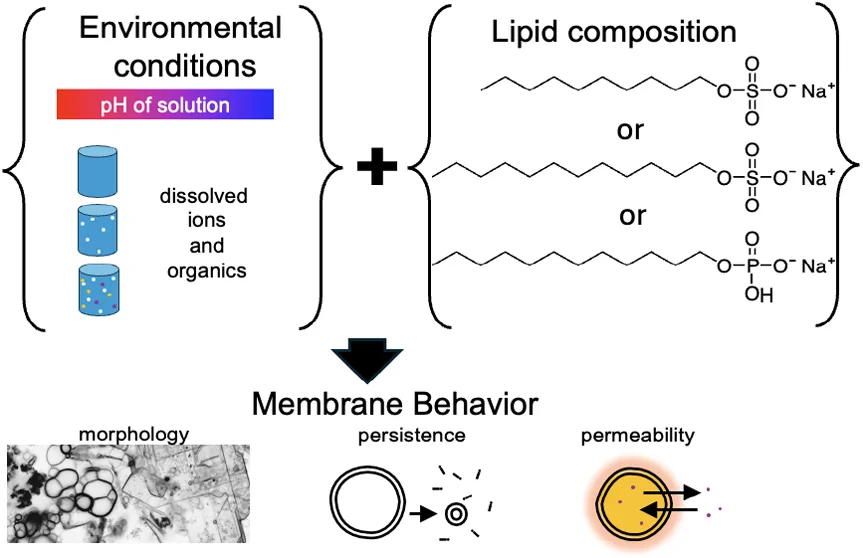

## Introduction

The origin of life on Earth remains one of the great scientific questions of our time.^1,2^ For life to emerge, the formation of individual cells using membrane compartments is considered an essential step.^3^ These structures, often described as protocells, could have provided an enclosed space conducive to the concentration and interaction of primitive biomolecules, paving the way for the first forms of metabolism and replication.^4,5^ Amphiphiles, characterized by a hydrophilic head and a hydrophobic tail, are found in interstellar and geochemical syntheses^6,7^ and can spontaneously self-assemble into micelles, bilayers, or vesicles depending on the physicochemical conditions of the environment.^8,9^ Several studies have shown that short-chain fatty acids, as well as their sulfated or phosphorylated derivatives, can form stable vesicles in variable environments.^10–13^ However, the stability and permeability of primitive membranes are known to be highly dependent on factors such as pH, ionic strength, and the presence of divalent ions or complex salts such as those found in sea salt.^13–16^

Therefore, a major challenge in the study of protocells is to understand how the chemical nature of amphiphiles (length of the carbon chain, nature of the polar group) and environmental conditions (pH gradients, ionic composition of the medium) influence the formation, stability, and dynamics of vesicles.^17,18^ These parameters are especially relevant as they reflect the heterogeneous environments of the early Earth, such as hydrothermal vents, intertidal zones, and volcanic lagoons, where chemical and pH gradients could play a catalytic role in molecular self-organization.^11,12,19,20^ Furthermore, many studies have shown that sulfate and phosphate are abundant in modern marine environments and sediments, and they were likely also prevalent in ancient iron-rich seawater. As such, they may have provided key chemical components relevant to the origin of life on Earth.^21–24^ On this basis, sulfate- and phosphate-containing amphiphiles have been widely used as model systems with strongly anionic headgroups to investigate how charge influences membrane behavior.

Amphiphiles such as decyl sulfate (DS), dodecyl sulfate (DDS), and dodecyl phosphate (DDP) are particularly relevant models for studying primitive membranes^10,25–29^ and are distinguished by their negatively charged polar group, which promotes water interactions and self-organization. The increase in the length of the carbon chain from decyl (10) to dodecyl (12) directly affects the melting temperature and solubility in water, which can change the stability and permeability of the bilayer.^20^ DDP is also of major biological importance as a prebiotic analog of membrane-forming phospholipids.^13^ These structural differences make DDP, DDS, and DS model systems for exploring how simple variations in the compositions of protocells could influence the dynamics and robustness of membranes at the origins of life.

Despite progress in understanding how simple amphiphiles self-assemble into membrane structures, most experimental studies have examined only a mixture of few amphiphiles at a time and typically under a narrow set of environmental conditions. Prior work has therefore provided valuable but highly simplified views of protocell membrane behavior, often focusing on binary mixtures, a single dominant amphiphile species, or a limited range of pH and salt concentrations^10–12,14,27,28^ While these studies demonstrated that short-chain fatty acids and derivatives can form vesicles, few^13,28,30^ capture the chemical complexity or environmental heterogeneity expected on the early Earth, where diverse amphiphiles likely coexisted and interacted within fluctuating freshwater, marine, and organic-rich settings.

To address these limitations, the present study investigates eight distinct multicomponent mixtures composed of seven different amphiphile headgroups, varying the identity and concentration of the anionic component (DS, DDS, or DDP). By evaluating membrane formation, stability, and permeability across three geochemically relevant media (ultrapure water, sea salt, and sea salt supplemented with a complex prebiotic organic soup) and multiple pH conditions, our experiments approximate a far more realistic range of environments expected in geological settings. To the best of our knowledge, this level of compositional and environmental diversity has not previously been explored, and it allows us to examine how hydrocarbon chain length, headgroup chemistry, and environmental variables influence vesicle formation, critical vesicle concentration, and membrane permeability in mixed amphiphile systems. The present work is intended to compare experimentally observable membrane properties rather than provide a complete physicochemical description of all intermolecular interactions governing self-assembly.^11–13^ Building on a substantial body of prior work that has established key principles of amphiphile self-assembly, the present study seeks to develop a more comprehensive framework for understanding how chemically diverse amphiphile mixtures, including components representative of prebiotic soups, could have supported stable protocell membranes on the early Earth.

Within this context, the present study compares the effect of carbon chain length and polar group identity on formation and stability using the number of vesicles formed, the critical vesicle concentration (CVC), and the permeability as metrics. We tested formation and stability in different media (pure water, simulated sea salt, and sea salt + prebiotic soup) at different pHs to assess the extent to which environmental conditions influence self-assembly and membrane dynamics. This work aims to better understand the relationships among the molecular structure, environmental conditions, and membrane stability and to provide experimental evidence that may shed light on the role of simple amphiphiles in the emergence of protocellular systems at the origin of life.

## Methods

### Meteoric Soup Preparation

A mixture of approximately 67 g of 114 organic compounds and several grams of mineral oil were combined with 1 L of ultrapure water and heated to dissolve the solids and the pH was adjusted to 7 using 10 M NaOH.^31^ (For complete list of organics, see the Supporting Information.) This mixture represents a laboratory analog of the heterogeneous organic mixtures that may have accumulated on early Earth.

### Sample Preparation

Eight amphiphile compositions were chosen based on their ability to form membranes in water.^27^ Each composition had a different concentration of six (6) different 10-carbon amphiphiles and either DS, DDS, or DDP. Individual amphiphile concentrations were set between 0 and 5 mM for all amphiphiles, except decanol and glycerol monodecanoate, which had a maximum concentration of 1 mM. These amphiphiles are not intended to represent exact prebiotic compositions but rather to serve as model systems to systematically investigate how headgroup chemistry influences membrane properties.

Stock solutions were prepared as 50 mM decanoic acid (Thermo Fisher Scientific, Agawam, MA, USA), decylamine (Thermo Fisher Scientific, Agawam, MA USA), and decyltrimethylammonium bromide (DTAB; TCI, Portland, OR, USA) used here as a model for permanently charged cationic amphiphile, in 70% ethanol. Primary and secondary aliphatic amines have been identified in carbonaceous meteorites such as Murchison.^32,33^

Decanoate was prepared from decanoic acid at 0.1 M NaOH. Stock solutions were prepared from the 50 mM and 10 mM solutions by dilution to 10 and 2 mM, respectively. The same process was followed to prepare 50, 10, and 2 mM decyl sodium sulfate (DS, Chem-Impex Int’l Inc., Wood Dale, IL, USA), dodecyl sodium sulfate (DDS, Thermo Fisher Scientific, Agawam, MA, USA), and dodecyl sodium phosphate (DDP, Thermo Fisher Scientific, Agawam, MA, USA) in ultrapure water. Decanol (Thermo Fisher Scientific, Agawam, MA, USA) was brought up in 70% ethanol at concentrations of 15 and 3 mM. Glycerol monodecanoate (TCI, Portland, OR, USA) was dissolved in 70% ethanol and brought to concentrations of 10 and 2 mM. Sea salt (Lake Products Company LLC, Florissant, MO, USA) was prepared according to the manufacturer’s instructions, for a final concentration of 35 g/L to mimic modern ocean water. Sea salt + soup solution was prepared by dissolving 35 g/L sea salt in a 25 g/L preprepared soup solution. Solutions of 0.1 M HCl and 0.1 M NaOH were prepared with ultrapure water.

Each of the eight amphiphile combinations were prepared with four acid/base conditions (60 μL of 0.1 M HCl, 30 μL of 0.1 M HCl, 15 μL of 0.1 M NaOH, and 30 μL 0.1 M NaOH) and three media conditions (no salt/ultrapure water, sea salt, and sea salt + soup) in two replicates for microscopy, for a total of (3 × 8 × 4 × 3 × 2) experiments. In our experiments, we added the same volume of HCl or NaOH to each sample so that we could directly compare vesicle formation behavior across DS, DDS, and DDP under the same chemical challenge. This way, every sample experiences the same absolute perturbation, letting us focus on how the headgroup and chain lengths affect membrane stability.

Samples were prepared using an Opentrons OT-2 liquid handling robot (Brooklyn, NY, USA) to dispense amphiphile stock solutions into 96-well SBS microplates. Reproducibility was checked using the protocol described in ref 27. After the dispensing process, samples were dried using a vacuum concentrator (SpeedVac SPD120, Thermo Scientific, USA) at 35 °C for 2 h 30 min. After all samples were rehydrated in 200 μL of appropriate aqueous environment, the SBS plates were placed on an OT-2 shaker for 30 min at 1000 rpm to obtain well-mixed homogeneous solutions.

### Critical Vesicle Concentration

Among the eight compositions, composition B in Figure 1, with the highest total amount of amphiphile, was selected for critical vesicle concentration (CVC) determination because of its ability to form vesicles in all environments. Quantitative CVC measurements require stable vesicle formation; therefore, only Composition B was suitable for this analysis, as it was the only composition that consistently formed vesicles under all tested conditions. Plates were prepared in duplicate with each sample having 21 dilutions from 0 to 100% in 5% intervals. To each 100 μL sample, 1 μL of 4 mM merocyanine 540 in ethanol was added. Visible absorbance was collected from 400 to 600 nm using a UV/vis plate reader Multiskan SkyHigh microplate spectrophotometer (Thermo Scientific, USA). The baseline correction was done by subtracting out at 600 nm, and normalization to the 530 nm peak across all samples was carried out to reduce media-dependent photophysical variation. The normalized intensity at 570 nm was then used to determine the CVC. The CVC value was then proportional to the ratio of intensity at 570 nm to intensity at 530 nm.^13,34^ Data are available at https://github.com/mfbliposome/Environmental_stability.

**Figure 1:**
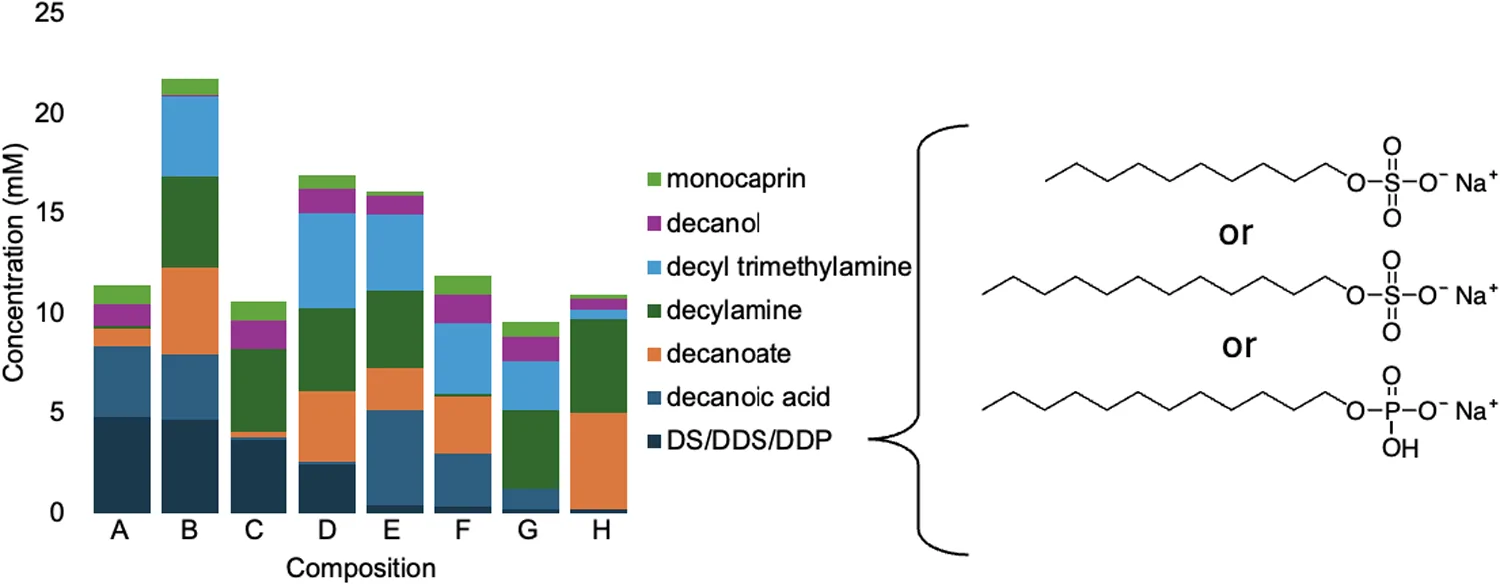
Eight amphiphile compositions. A–H are listed from the highest concentration of variable anionic amphiphile to the lowest concentration.

### Membrane Permeability

Composition B was further evaluated for membrane permeability (Figure 1). As with the CVC measurements, quantitative permeability analysis requires stable vesicle formation; therefore, only Composition B was used, since it was the only composition that consistently formed vesicles under all tested conditions. Fluorescent vesicles were prepared by rehydrating the dried composition with 30 μM NBD-DOPE (1,2-dioleoyl-*sn*-glycero-3-phosphoethanolamine-*N*-(7-nitro-2–1,3-benzoxadiazol-4-yl) ammonium salt, Avanti Polar Lipids, USA) in 50 mM HEPES and 100 mM NaCl buffer at pH 7.2. Control samples were prepared identically but with the addition of 0.2% Triton X-100 (Fisher, USA) to form micelles, providing complete exposure of the dye to quenching.

Fluorescence measurements were performed using a ThermoScientific Varioskan Lux (Thermo Fisher Scientific, Waltham, MA, USA) microplate reader with an integrated reagent dispenser. Dithionite (10 μL of 100 mM in HEPES buffer) was added to each well and mixed at 1000 rpm. Dithionite reacts with NBD-DOPE to irreversibly quench fluorescence, but it cannot readily cross intact vesicle membranes. Therefore, the rate of fluorescence decay over time reflects the rate of dithionite permeation through the bilayer. Fluorescence was read every 10 ms over 600 scans for 30 min. Fluorescence decay curves were modeled using a system of differential equations,^35–37^ from which the permeability rate constant, *k*(s^–1^), was determined for vesicles containing DS, DDS, or DDP. The experimental design compares composition B differing only in the inclusion of DS, DDS, or DDP, with all permeability measurements performed under identical buffer conditions (50 mM HEPES, pH 7.2). All membrane compositions were evaluated under the same conditions to understand the differences in permeability in a single environment with changing composition. Therefore, the observed differences in permeability reflect differences in amphiphile identity and membrane organization under these specific environments to provide insights into permeability of different compositions on early Earth.

### Confocal Microscopy

Using the OT-2, 10 μL of Nile Red was added to 100 μL of each sample; then, 50 μL of each sample was transferred from a 96-well plate to a 384-well plate fitted with a coverslip base (Cellvis, USA) for automated confocal microscopy. Samples were prepared in duplicate and imaged using a Nikon AXR confocal microscope with a 60×/1.40 Plan Apo Lambda D oil immersion lens and 561 nm excitation. A custom routine for automated 384 well-plate imaging was programmed using NIS Elements’ JOBS function. Each well was imaged from four random fields and exported as a TIFF file. The raw microscopy data are available at 10.5281/zenodo.17612643.

## Results and Discussion

This work compared the formation of vesicles in a variety of environments relevant to the origins of life. We evaluated these mixtures in ultrapure water (no salt) to mimic fresh water environments, such as ponds, a salt mixture to mimic the ionic composition of oceans, and salt and organics (salt + soup) to mimic an ocean with dissolved organics from interstellar and terrestrial sources. The eight amphiphile mixtures (A–H, Figure 2) were also selected to represent a “messy chemical system” that we would expect in abiotic environments. From previous work, decyl sulfate concentration in a seven-component mixture was found to promote vesicle formation. Decyl sulfate (DS), dodecyl sulfate (DDS), and dodecyl phosphate (DDP) were compared to understand the role of both the headgroup and tail length in membrane formation in these environments.

**Figure 2:**
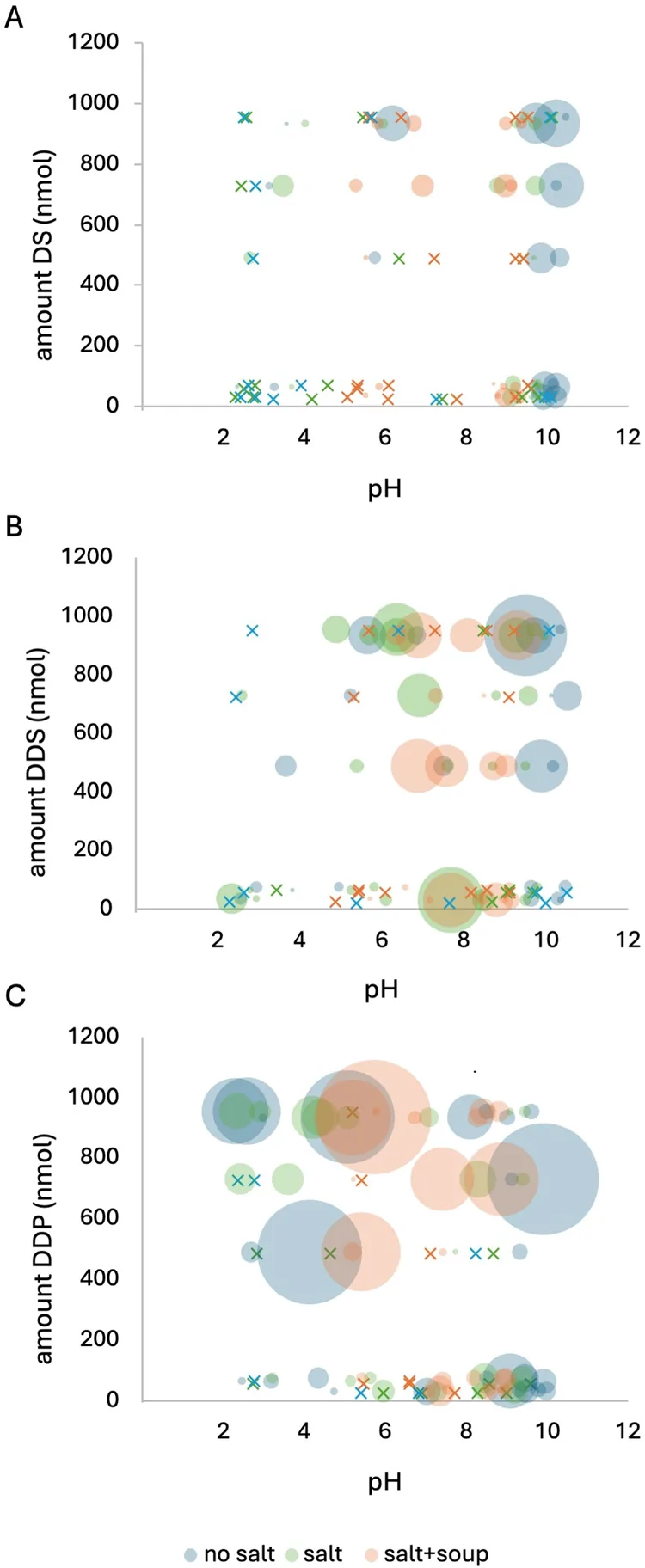
Vesicle formation by pH and amount of anionic amphiphile. Decyl sulfate (DS, A)-, dodecyl sulfate (DDS, B)-, and dodecyl phosphate (DDP, C)-containing samples are compared by aqueous solution. Samples were prepared in ultrapure water (no salt, blue), sea salt (salt, green), or sea salt and organics (salt + soup, orange). The circle area is relative to the number of vesicles counted in the sample; x is a sample that did not form membranes.

All compositions were evaluated for vesicle formation at four acid/base conditions and three solution conditions. We found that the composition with the highest total amphiphile composition, B, was the most successful at forming vesicles. Composition B also had a very high concentration of the anion being tested (DS, DDS or DDP, referred to as the “anionic surfactant”), with about equal amounts of the other amphiphiles, excluding decanol. Composition B performed the best in the moderate pH water environment.

The other compositions that formed vesicles in some environments include A, C, and D. Compositions A and C were mostly anionic surfactants and either decanoic acid or decylamine, respectively. Composition A heavily favored forming vesicles with DDP, while composition C was successful when either DDP or DS was present but interestingly not in compositions containing DDS. Composition D was a mixture of anionic surfactants, decanoate, decylamine, and DTAB. Both DDS- and DDP-containing compositions formed membranes, with DDP favoring the lower pHs. DS-containing compositions were only successful vesicle formers in the higher pH water samples. These observations demonstrate the challenge of the compositional prediction of vesicle formation in different environments.

The compositions with the least anionic surfactant content, E–H, were less successful vesicle formers. Compositions E and F were similar: they were mostly made of decanoic acid, decyl amine, and DTAB. Composition G was mostly a cationic surfactant, with 67% of the amphiphiles as either decylamine or DTAB. There was almost no vesicle formation in any of these compositions regardless of the environment. Composition H was unique in that it had the lowest amount of anionic surfactant, yet the outcomes for each type of ionic surfactant were different. Very low, but persistent levels of vesicle formation (<10 vesicles counted) were seen in many of the environments with the DDP compositions. With DS, only the NaOH environments formed the membranes. In the DDS compositions, many vesicles were found apart from the water environments. These findings indicate that the compositions that contain DTAB but not the anionic surfactant appear unstable, possibly because the positive charge cannot be countered by the other amphiphiles present, allowing dissolution or micellization to occur.

The number of vesicles in each of the (3 × 8 × 4 × 3 × 2) samples was evaluated to assess membrane formation in environments that varied in pH, the amount of DS/DDS/DDP, and dissolved aqueous species (Figure 2). Samples with more DS, DDS, or DDP, as indicated by the *y*-axis, generally had more vesicle counts per micrograph (larger circles). Low-pH samples had smaller vesicle counts than higher pH samples, especially in the sulfate headgroup samples.

How does increasing the hydrocarbon chain length from 10 to 12 impact the vesicle formation? Decyl sulfate (DS, Figure 2a) had the fewest vesicles per micrograph and the most samples without vesicles (46% compared to 31% in DDS or 24% in DDP). Shorter hydrocarbon chains are generally more soluble in water and therefore need more material to aggregate into membranes, which could explain why DS compositions had fewer samples with vesicles and fewer vesicles in those samples. The most vesicles for DS were found in samples at a high pH in water. In comparison, DDS had samples with many vesicles at pHs of <6 across all aqueous conditions. The stability imparted by DDS also extended to samples that had very small amounts of DDS added (<2% of the composition), with 29 samples having some vesicle formation in these samples compared to only 22 in the DS-containing compositions.

Figure 3a summarizes the results for the 8 × 4 × 3 samples. Among them, 18 samples did not form vesicles with either DS or DDS. In contrast, 20 samples formed vesicles with DDS but not with DS, while 13 samples showed the opposite behavior, forming vesicles with DS but not with DDS. The remaining 45 samples formed vesicles under both DS and DDS conditions. To quantify this comparison, samples were grouped according to whether their behavior agreed or differed between the two systems, and the corresponding percentages were calculated over the 96 DS and 96 DDS samples. 63 samples exhibit the same behavior, while 33 differ.

**Figure 3:**
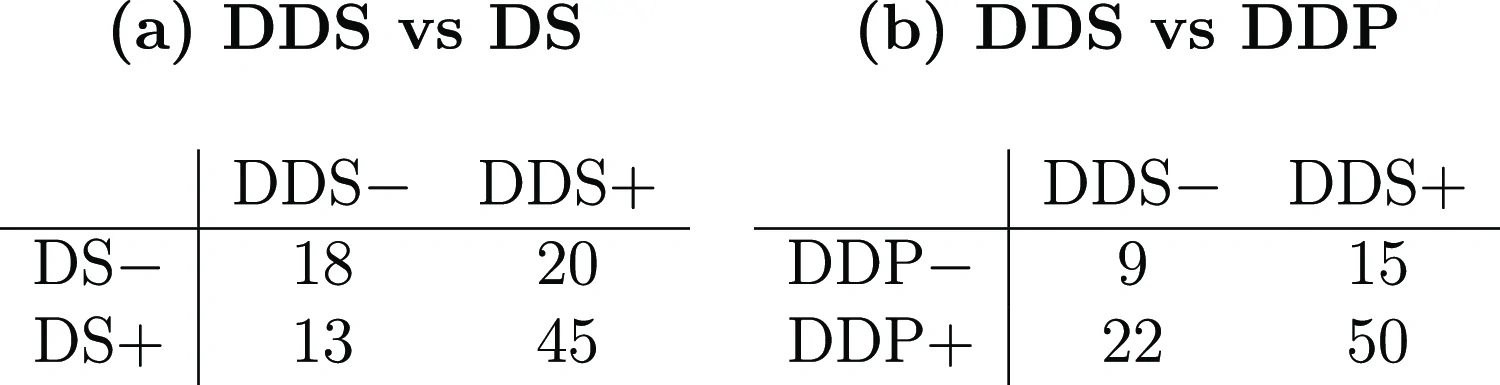
Confusion matrices comparing vesicle formation outcomes for anionic surfactants. Vesicle presence (+) or absence (−) for DDS is shown along the columns and for (a) DS and (b) DDP along the rows. Diagonal elements indicate agreement in vesicle formation behavior.

Confusion matrix analysis (Figure 3a) shows that DS and DDS vesicle formation behavior agrees in approximately 66% of the tested conditions, indicating partial similarity between the two systems. However, the remaining 34% of conditions show disagreement, underscoring that changes in the chain length significantly shift the environmental thresholds for vesicle formation and persistence. These results suggest that increasing the chain length alone does not guarantee enhanced membrane stability but instead modulates the sensitivity of vesicle formation to environmental parameters such as pH and ionic strength.

How does changing the headgroup from sulfate to phosphate modify vesicle formation? Generally, the phosphate-containing compositions (Figure 2c) generated the most countable vesicles (the circle area represents the vesicle count). Phosphate samples were slightly lower in pH compared to the dodecyl sulfate samples (Figure S2, Supporting Information). The low-pH samples containing phosphate headgroups were excellent vesicle formers (Figure 4) and had samples forming vesicles at pH as low as 2.28.

**Figure 4:**
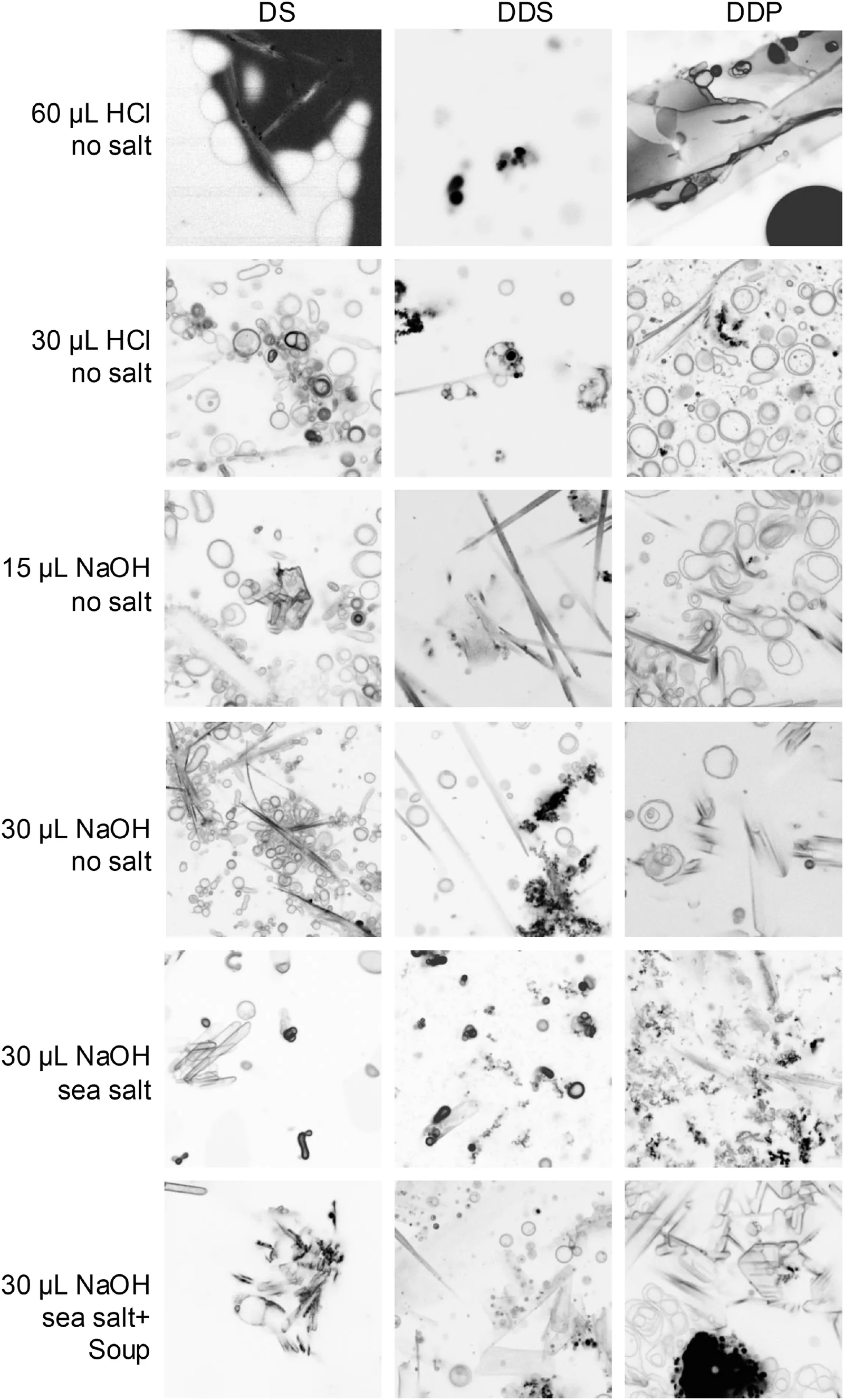
Confocal micrographs of the sample morphology. Each column has a different anion present in the liposome composition. Each row is a different environment as listed on the left to compare no salt with differing pH and the high pH with three dissolved solute conditions. Each micrograph is 48 μm-wide, stained with Nile Red dye. Image colors are black/white inverted in this figure.

Figure 3b shows the results for the 8 × 4 × 3 samples. Among them, nine samples did not form vesicles with either DDS or DDP. In contrast, 15 samples formed vesicles with DDS but not with DDP, while 22 samples formed vesicles with DDP but not with DDS. The remaining 50 samples formed vesicles under both DDS and DDP conditions represent the majority of agreement.

Confusion matrix analysis (Figure 3b) indicates that DDS and DDP agree in approximately 61% of cases, with disagreement observed in 39% of samples. Notably, DDP forms vesicles in 22 cases, whereas DDS does not, suggesting a broader stability range window. This enhanced robustness is consistent with phosphate headgroups conferring greater membrane stability under diverse environmental conditions. These results highlight the role of phosphate headgroups in enhancing membrane robustness and emphasize their relevance in early protocell models.^13,28^

Representative micrographs of Composition B are shown in Figure 4. All micrographs can be accessed at 10.5281/zenodo.17612643. Composition B had vesicles forming under most environmental conditions; however, a large variation in morphologies can be seen: oil is shown as black spots and crystal-like needle shapes are visible, along with thin bubbles that represent unilamellar or multilamellar membranes. Here, the crystal phases must have some hydrophobic character (i.e., not simply inorganic crystals) because these are visualized using Nile Red dye that is only fluorescent when in hydrophobic environments. Acidic environments have more oily phases (top row) compared to basic environments in water. The presence of sea salt, either with or without dissolved organics, increased the number of nonvesicle phases. The present work focuses specifically on vesicle formation because membrane compartments are the structures most relevant to protocell models. While it is possible that aggregates such as oil-rich phases and crystalline structures partition specific amphiphiles from the vesicle phases, the observation that protocells are still present with such a composition/environment pair is important to understanding the persistence of protocells in these complicated mixtures. The detailed physicochemical characterization of these nonvesicle aggregates was beyond the scope of this study and will require complementary techniques in future work. Other compositions (A and C–H) had different morphological behaviors that result from the specific compositions (see the Supporting Information).

Figure 5 shows the variation of the critical vesicle concentration (CVC) in composition B as a function of environmental conditions (no salt, salt, salt + soup) for the three groups of amphiphiles represented by their polar heads: DDP, DDS, and DS. Bars indicate the average CVC for different amounts of added acid or base (NaOH or HCl). DDP generally shows a higher CVC, particularly in the presence of 30 μL of 0.1 M NaOH without salt, suggesting that samples with the phosphate headgroup are more sensitive to the alkalinity of the medium. The CVC of DDS also increases under some basic conditions but less so than that of DDP, reflecting an intermediate pH sensitivity. DS maintains relatively low CVC values regardless of the environment, indicating greater robustness or less sensitivity to environmental variations. Although Merocyanine 540 can exhibit pH and salt-dependent photophysical variation, the normalization strategy used here reduces these interferences, supporting that the observed CVC reflects the difference among the amphiphile compositions rather than the dye alone. Interestingly, in fatty acids, for example, longer hydrocarbon chains are less soluble in water and therefore thought to lower CVC.^17^ However, in these mixtures, where less than 20% of the molecules are changing, this trend does not hold true. Other properties of the longer amphiphiles may have contributed negatively to the CVC. It is possible that the higher gel to liquid transition temperatures of the longer amphiphiles or the mismatch in chain length between the bulk composition, which are all 10-carbon amphiphiles, and the DDS or DDP causes instability in these mixtures.

**Figure 5:**
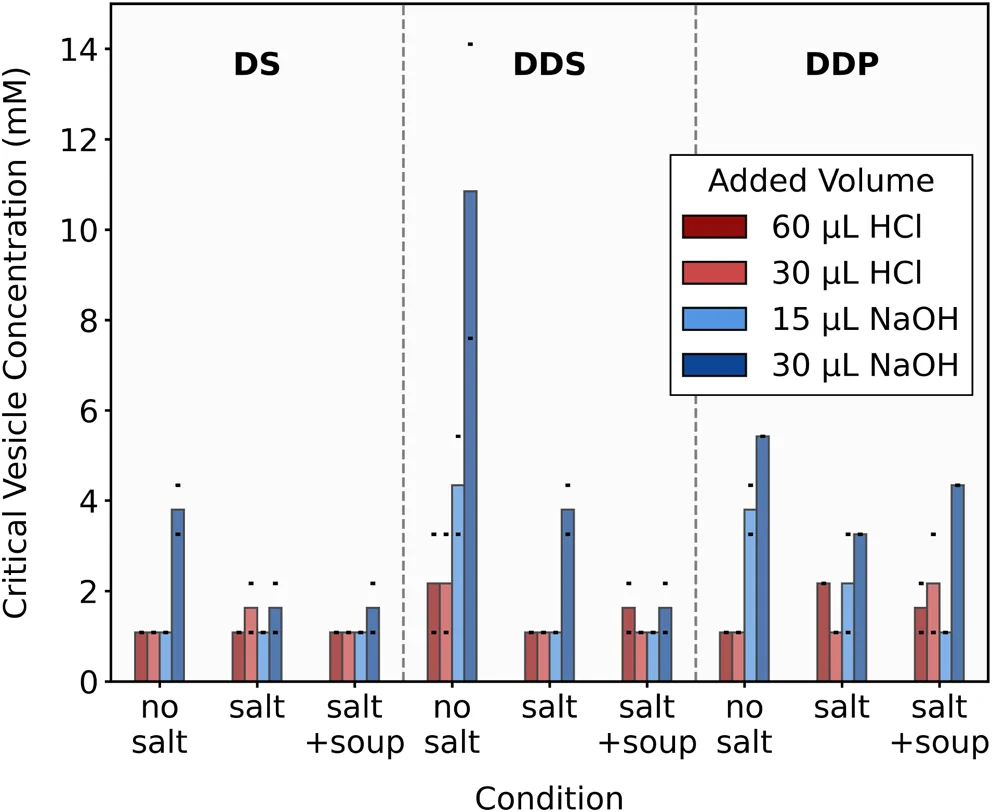
Critical vesicle concentration of composition B in all environments. Clustered by anionic species from left to right: DDP, DDS, and DS. Further grouping by cosolutes: none (ultrapure water), 35 g/L sea salt, or 35 g/L sea salt + 25 g/L dissolved organics. Colors indicate acid or base addition. CVC was determined in duplicate as represented by black lines.

Overall, the presence of sea salt tends to reduce CVC, probably by changing the solubility of hydrophobic moieties, which has been observed previously.^34^ There are also possible effects of salt interactions with the charged headgroups. The effect of organics on CVC was negligible in this experiment. The addition of organics did not increase the CVC of the sea salt samples, which were already at the minimum concentration tested, reinforcing the idea that these membranes are generally stable in messy prebiotic systems.

The contrasting behaviors of DDP and DDS show that the chemistry of the polar head plays a central role in the self-organization. This, in turn, suggests that molecular diversity may have helped support the emergence of early protocellular membranes.

Composition B was also evaluated for its membrane permeability. The dried mixture was rehydrated with NBD-tagged DOPE in a buffer to form fluorescently active vesicles. The kinetics of fluorescence decay were determined by treating the vesicles with a strong reducing agent, dithionite. The fluorescence decay was determined for composition B with either DS, DDS, or DDP in the composition (Figure 6). We also tested vesicles composed of pure decanoic acid (C10:0) and oleic acid (C18:1).

**Figure 6:**
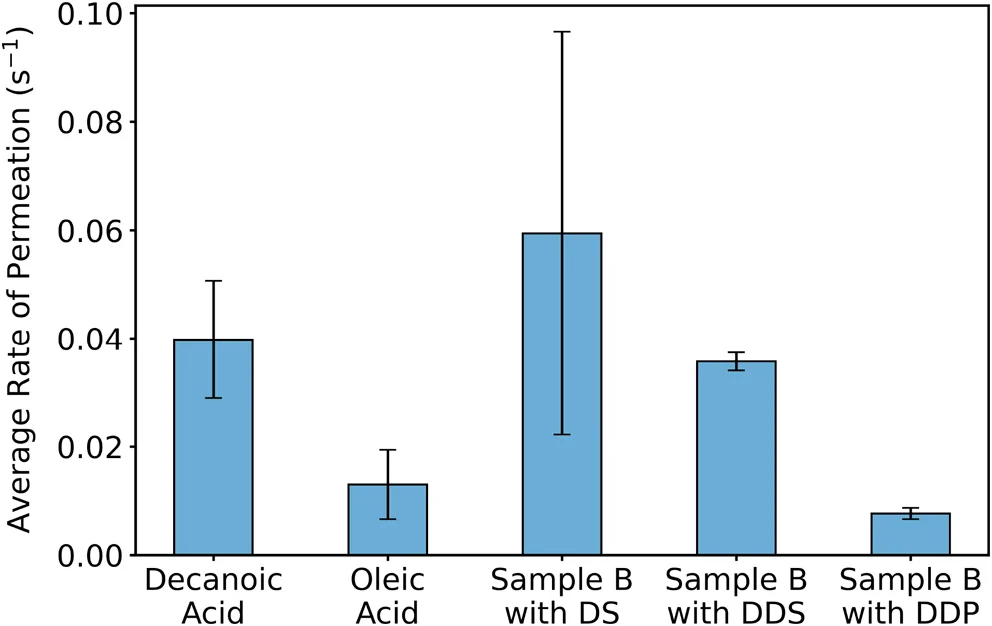
Average rate of permeation for different lipids.

The average membrane permeability varies significantly across different lipid compositions with smaller *k* values indicating a slower rate of permeation. Vesicles with DDP exhibited the lowest permeation rate (0.00765 s^–1^), indicating that phosphate-containing membranes are less permeable to dithionite. This reduced permeability is consistent with the strong hydration and hydrogen-bonding capacity of phosphate headgroups, which can increase interfacial cohesion and lead to tighter membrane packing.^38,39^ The protonation state of the phosphate headgroup in our samples was not determined. However, we expect that the acidic proton on the phosphate headgroup is stabilized within the membrane environment, resulting in an apparent p*K*_a_ higher than its dilute solution value (about 7), as has been observed for other amphiphiles such as oleic acid,^14,40^ and therefore, we would expect it to be protonated in these measurements. Vesicles containing sulfate headgroups (composition B with DS or DDS) showed higher permeation rates consistent with greater solute diffusion.^13,41^ Vesicles of longer-chain length amphiphiles were less permeable than those composed of shorter amphiphiles when comparing fatty acid and alkyl sulfates.^13,28,30^ This behavior likely reflects weaker hydration and increased solute diffusion across the membrane interface in sulfate-containing systems.

Overall, these results suggest that headgroup chemistry strongly influences the membrane stability and permeability in these simple lipid systems. These effects point to a combined influence of headgroup hydration, electrostatic interactions, and lipid packing on membrane permeability.^42^ It is possible that some of the differences in permeability are related to differences in surface charge between the compositions containing phosphate versus sulfate due to the phosphate having a second acidic proton. However, we expect the phosphate proton to be stabilized by aggregation, similar to that for fatty acids, and therefore, at the pH measured, it is also possible that the surface charges are very similar to the sulfate-containing vesicles and therefore not contributing to permeability differences. Regardless of surface charge, we have determined that at neutral pH, the phosphate headgroup-containing membranes are less permeable than sulfate headgroup-containing amphiphiles.

## Conclusions

Membrane formation from multicomponent amphiphile mixtures is strongly dependent on environmental conditions. In this study, vesicle formation and persistence varied with pH, ionic strength, and composition, highlighting the sensitivity of these systems to their chemical environment. Across all mixtures, vesicle formation was generally favored at higher pH and reduced under acidic conditions, with sulfate-containing systems showing greater sensitivity. Headgroup chemistry played a key role in determining membrane behavior. Phosphate-containing mixtures formed vesicles across a broader range of conditions compared to sulfate-based systems. Differences in hydrocarbon chain length shifted the environmental boundaries for vesicle formation but did not lead to a simple increase in stability. A detailed analysis of composition B showed that CVC values were low across many conditions, while permeability measurements revealed that DDP-containing membranes were less permeable to dithionite than sulfate analogs. This reduced permeability, combined with strong environmental responsiveness at higher pH, suggests that phosphate-containing amphiphiles can form membranes that are both stable and tunable under changing conditions. Overall, these results demonstrate that membrane formation, critical vesicle concentration, and permeability vary systematically with amphiphile composition and environmental conditions. While the present work does not directly quantify all molecular interactions responsible for these effects, the observed trends are consistent with contributions from headgroup chemistry, membrane packing, hydration, and electrostatic interactions. Rather than following simple trends observed in single component systems, these mixtures display more complex and context-dependent behavior. Such coupled effects are likely to be important in understanding how simple membranes assemble and persist in heterogeneous aqueous environments. Recent predictive models have shown success in estimating liposome formation under fixed conditions, and our results highlight that environmental factors such as pH and ionic strength strongly influence vesicle formation, suggesting that including such variables in future predictive models could improve their relevance to real systems.^43–45^ Future studies incorporating complementary physicochemical measurements, including surface charge characterization and detailed phase analysis, will further clarify the molecular mechanisms underlying these observations.

## Supplementary Material



## Data Availability

Microscopy image data are available on Zenodo at 10.5281/zenodo.17612643. Other data and code in this work are available on Github at https://github.com/mfbliposome/Environmental_stability and https://github.com/mfbliposome/opentrons.

## Notes

Experimental details and characterization data, including: a description of the Meteoric Soup section with Table S1 summarizing the abundance of organic compounds identified in the Murchison meteorite; pH comparison data for DS, DDS, and DDP systems, including Figure S1 showing pH comparisons between DS, DDS, and DDP and Figure S2 presenting the average pH values of samples containing DS, DDS, and DDP headgroups under different environmental conditions; confocal micrographs showing vesicle morphology for different lipid compositions, including Figure S3 for composition A (with each column representing a different anion present in the vesicle composition), Figure S4 for composition C, Figure S5 for composition D, Figure S6 for composition E, Figure S7 for composition F, Figure S8 for composition G, and Figure S9 for composition H; and membrane permeability measurements, including permeability assays of composition B and reference lipid systems (decanoic acid, oleic acid, and DOPC), fluorescence decay curves, kinetic fitting analyses, residual plots, and details of the data processing and modeling methods used for permeability calculations (PDF)

## Abbreviations

CVC: critical vesicle concentration
DS: decyl sulfate
DDS: dodecyl sulfate
DDP: dodecyl phosphate
