# Supplementary material for: Environmental Stability of Alkyl Phosphate- and Sulfate-Containing Membranes as Early Cell Models
Source: ACS Earth Space Chem. 2026 Sep 24;10(10):2310–7. doi: 10.1021/acsearthspacechem.6c00171 (PMC13679080; doi:10.1021/acsearthspacechem.6c00171)
Supplement: Supplementary file 1 [file sp6c00171_si_001.pdf]

Supporting Information: Environmental Stability of Alkyl Phosphate and Sulfate-Containing Membranes as Early Cell Models

Christelle Ekosso,† Hao Liu,‡ Avery Glagovich,† Adam Rogers,† Lila N. Strober,¶ Joshua Schrier,*,‡ and Sarah Maurer*,†

†*Department of Chemistry and Biochemistry, Central Connecticut State University, 1615 Stanley Street, New Britain, Connecticut, 06050, USA*

‡*Department of Chemistry and Biochemistry, Fordham University, 441 East Fordham Road, The Bronx, New York, 10458, USA*

¶*Department of Chemistry, Tufts University, 419 Boston Ave, Medford, Massachusetts, 02155,USA*

E-mail: jschrier@fordham.edu; smaurer@ccsu.edu

Contents

1. Meteoric Soup **4**

2. pH Comparision **4**

3. Micrograph morphology **7**

4. Membrane Permeability **9**

List of Figures

S1 pH comparison between DS, DDS and DDP 6
S2 Average pH of samples with DS, DDS and DDP head group in all environments. 7
S3 Confocal micrographs of sample morphology with composition A. Each column has a different anion present in the vesicle composition. 10
S4 Confocal micrographs of sample morphology with composition C. 11
S5 Confocal micrographs of sample morphology with composition D. 12
S6 Confocal micrographs of sample morphology with composition E. 13
S7 Confocal micrographs of sample morphology with composition F. 14
S8 Confocal micrographs of sample morphology with composition G. 15
S9 Confocal micrographs of sample morphology with composition H. 16

List of Tables

S1 Abundance of organic compounds in Muchison Meteorite. From[1] 5

1. Meteoric Soup

The meteoric soup was prepared in water from commercially available organics. The relative abundance of each organic was determined from the natural abundance of organics in the well characterized carbonacious Murchison Meteorite. Table S1 shows the abundace of chemical groups within the samples. Within each chemical group, only species in greater than 10 nmol/g meteorite were used. For example, there were 20 amines detected in Murchison samples, but only ethylamine and methylamine were greater than our threshold of 10 nmol/g at 18 and 71 nmol/g respectively. The class of amines was 1.09% of the Murchison organics so we made the sum of methylamine and ethylamine 1.09% of the organics used in our "soup" mixture. An exception was made for "aliphatic and aromatic hydrocarbons" where a small amount of mineral oil was added to represent a diversity of these non-soluble compounds.

A full list of organics and references can be found in the file: `https://github.com/mfbliposome/Environmental_stability/tree/main/soup`

2. pH Comparision

The pH was measured for all samples. A comparison of pH when using decylsulfate (DS), dodecylsulfate (DDS) or dodecylphosphate(DDP) is shown in Fig. S1. In NaOH adjusted samples, DS compositions were higher in pH than the DDS composisions. In HCl ajusted samples, the opposite is seen with lower pH in the DS compositions when compared to the DDS compositions. The use of DDP, compared to DDS caused lower pH values in general with the exception of Composition H which had the lowest concentration of these amphiphiles (0.18 mM).

The heatmap shown in Fig. S2 gives a clear picture of how the pH changes under different conditions for the three head groups. As expected, the samples with NaOH are much more basic, while those with HCl become strongly acidic. Across most conditions, DDS and DS

Table S1: Abundance of organic compounds in Muchison Meteorite. From[1]

compound class	ug/g	percent of total
Aliphatic and aromatic hydrocarbons		6.12
Aliphatic hydrocarbons*	23.5	3.20
Aromatic hydrocarbons*	21.5	2.92
Polar Hydrocarbons	38	5.17
monocarboxylic acids	232	35.10
dicarboxylic acids	53	7.97
amino acids	60	8.16
hydroxy acids	14.6	1.99
dicarboximides†	50	6.89
pyridine carboxylic acids‡	67	9.31
sulfonic acids	57	0.10
phosphonic acids	1.5	0.20
n-heterocycles*	0.275	0.04
amines	8	1.09
amides	trace	
polyols	60	8.16
purines	1.2	0.16
urea	25	3.40

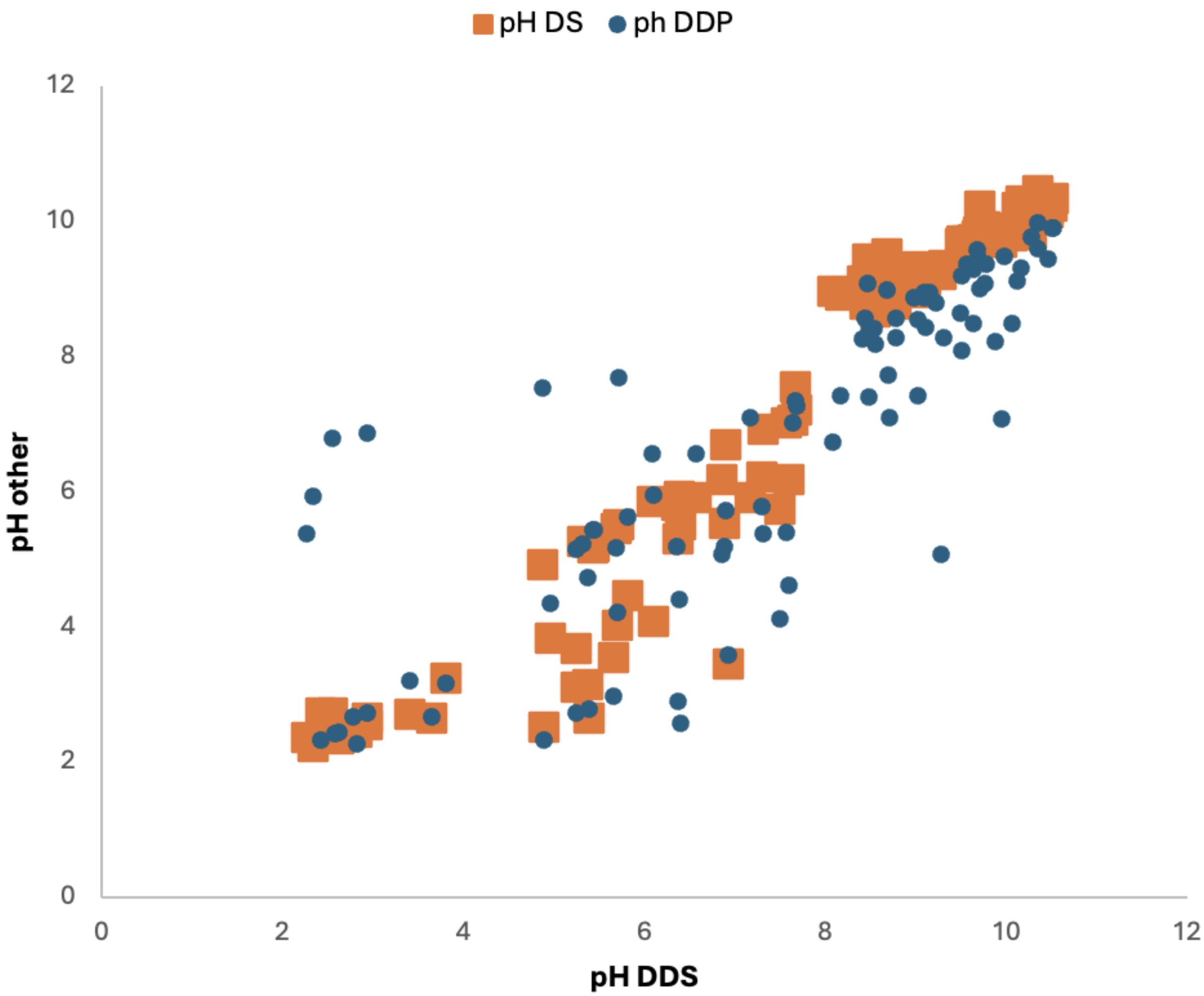

Figure S1: pH comparison between DS, DDS and DDP

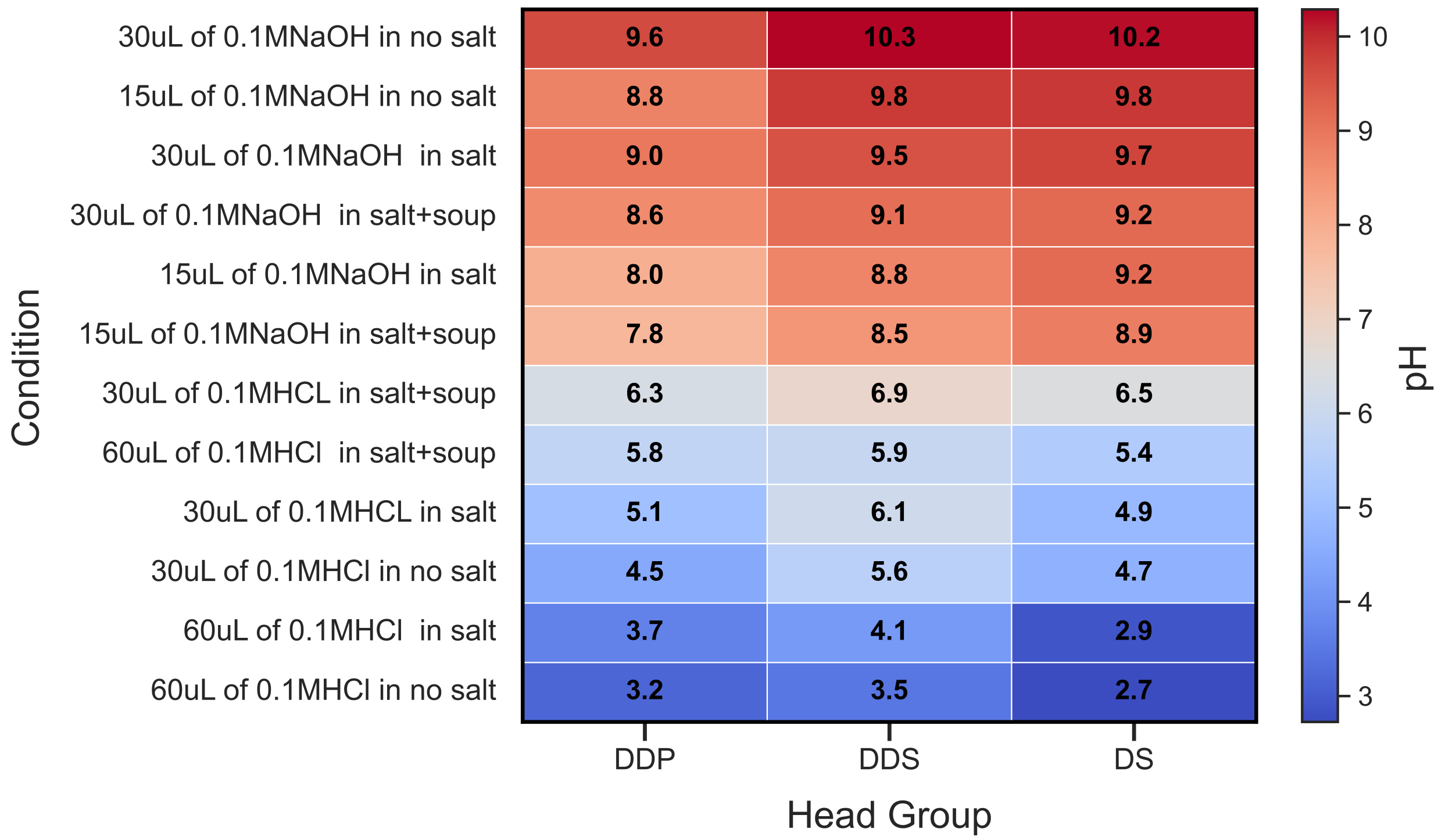

Figure S2: Average pH of samples with DS, DDS and DDP head group in all environments.

tend to reach slightly higher pH values than DDP. Adding salt changes the pH slightly trending more toward neutral, which is possibly an effect of changing the pKa of the weakly acidic amphiphiles, or the activity of water, or a changing pH electrode response in the presence of salt. The organic soup contains some weak acids like hydroxy and amino acids, as well as phosphate and therefore acts as a buffer for these environments. The figure also reiterates the head group response to the different environments with lower pHs in DDP compositions. Data are available at `https://github.com/mfbliposome/Environmental_stability/tree/main/Data/20250307`

3. Micrograph morphology

Compositions A and C-H exhibit distinct morphological behaviors across the explored conditions, with each composition showing a characteristic response depending on the head group

(Fig S3-S9). Note that Composition B is in the main text. We chose to display here only no salt samples for all pHs conditions and the high pH samples with salt and salt+soup for comparison. Compositions are organized from highest to lowest amount of DS/DDS/DDP alphabetically, A through H. Across all compositions, many samples have non-vesicle phases, as precipitated solids or crystals. This set of micrographs highlights the high amount of heterogeneity in these samples even when vesicles are forming, with crystals, and other solid objects often appearing with the vesicles in a single sample. This work also demonstrates the need for greater environmental diversity when designing experiments to test hypotheses about self-assembly processes for the origins of life: small changes in composition, pH and ionic strength result in different morphologies that are not easy to predict.

DDP appears to have more solids in most environments. By contrast, vesicle-like compartments emerge under basic conditions with no salt, with DDP in particular forming well defined multilamellar structures, an effect that is especially pronounced under prebiotic saline conditions. Composition C in Fig. S4 shows vesicle formation only under basic conditions with no salt, and this behavior is observed for both the DS and DDP head groups. In Fig. S5, composition D shows little to no vesicle formation across all tested head groups and conditions. In Fig. S6, Composition E supports vesicle formation for DDS and DDP, whereas DS fails to assemble into vesicular structures. Under saline conditions and in the presence of prebiotic soup, however, only sparse or poorly defined structures are observed with DDP, indicating a strong suppression of organized assembly.A comparable trend is observed for composition F (Fig. S7), which gives rise to well defined structures in moderate basic environments across all head groups. However, the presence of salt and prebiotic soup largely suppresses assembly, with DDS being the notable exception.Composition G in Fig. S8 shows some self-assembled membranes under moderately acidic conditions for the DDS and DDP head groups. No head groups form structures under basic conditions in the absence of salt. The presence of salt appears to promote structure formation for DS, while salt

combined with prebiotic soup seems to have a positive effect on the DDP head group. In Fig. S9,composition H exhibits minimal vesicle formation, observed only under highly basic conditions for the DDS head group, both in the absence of salt and in the presence of salt with prebiotic soup; the other head groups do not favor membrane formation.

4. Membrane Permeability

Membrane permeability was measured for composition B as well as standard lipids: decanoic acid, oleic acid, and DOPC. The membrane permeability was not found for DOPC because the differential equations assume first order kinetics, which does not account for a membrane as stable as DOPC; DOPC did not allow enough dithionite to cross the membrane to entirely reduce the interior NBD during the course of the run. The graphs of fluorescence vs time for all lipids tested were included alongside numerical or fit data with the corresponding residuals. The fit equations were derived: `https://github.com/mfbliposome/Dithionite-Assays/blob/c2a43107112cdff989a5326c54264466f001de95/code/notebooks/Fluorescence_decay_modeling.ipynb` The fluorescence data was processed: `https://github.com/mfbliposome/Dithionite-Assays/tree/c2a43107112cdff989a5326c54264466f001de95/results` In this folder, only the date and lipid name indicate a unique sample. Several versions of analyzed data were generated from testing different processing methods.

References

(1) Sephton, M. A. Organic compounds in carbonaceous meteorites. *Natural Product Reports* **2002**, *19*, 292–311.

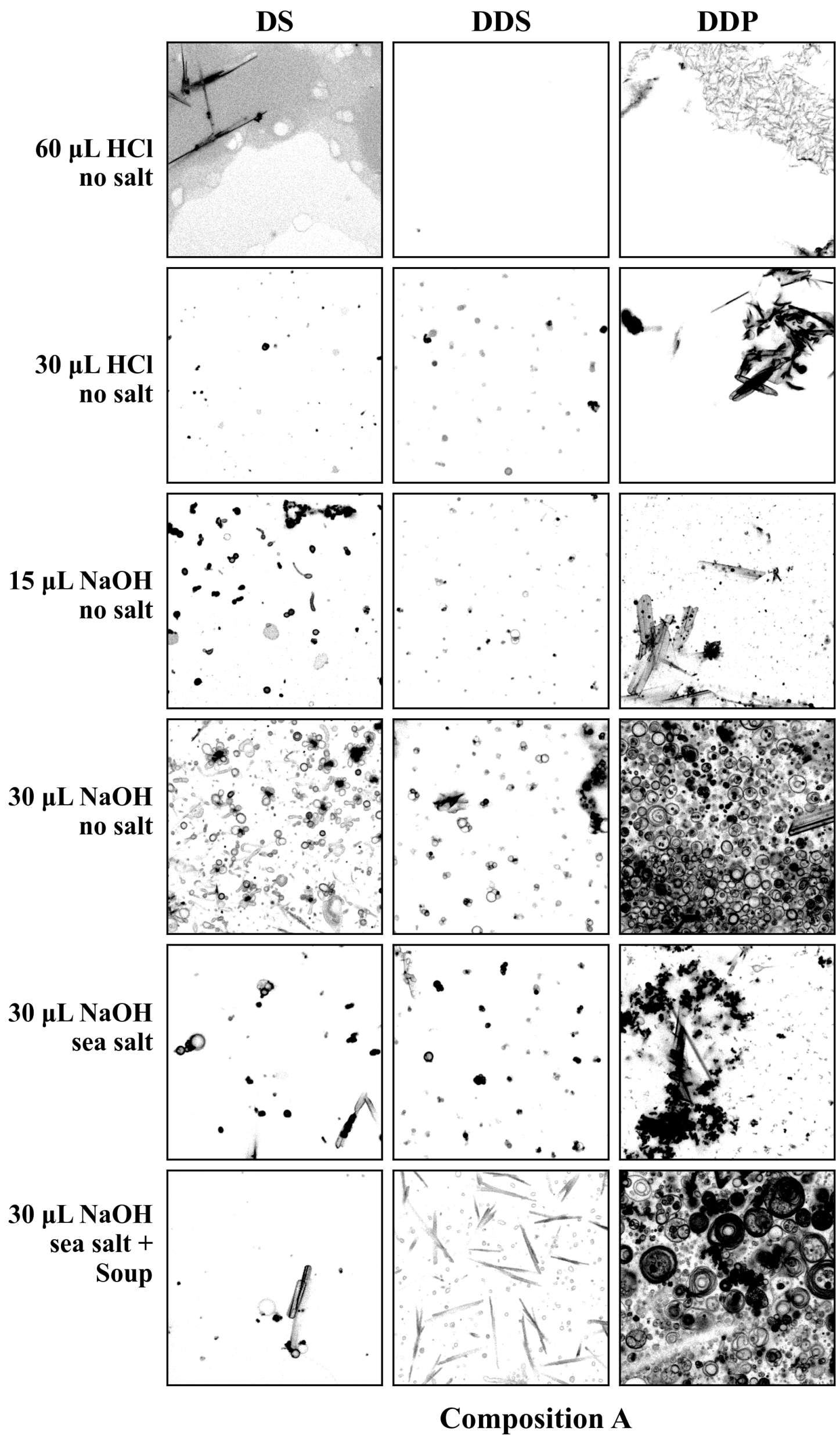

Figure S3: Confocal micrographs of sample morphology with composition A. Each column has a different anion present in the vesicle composition.

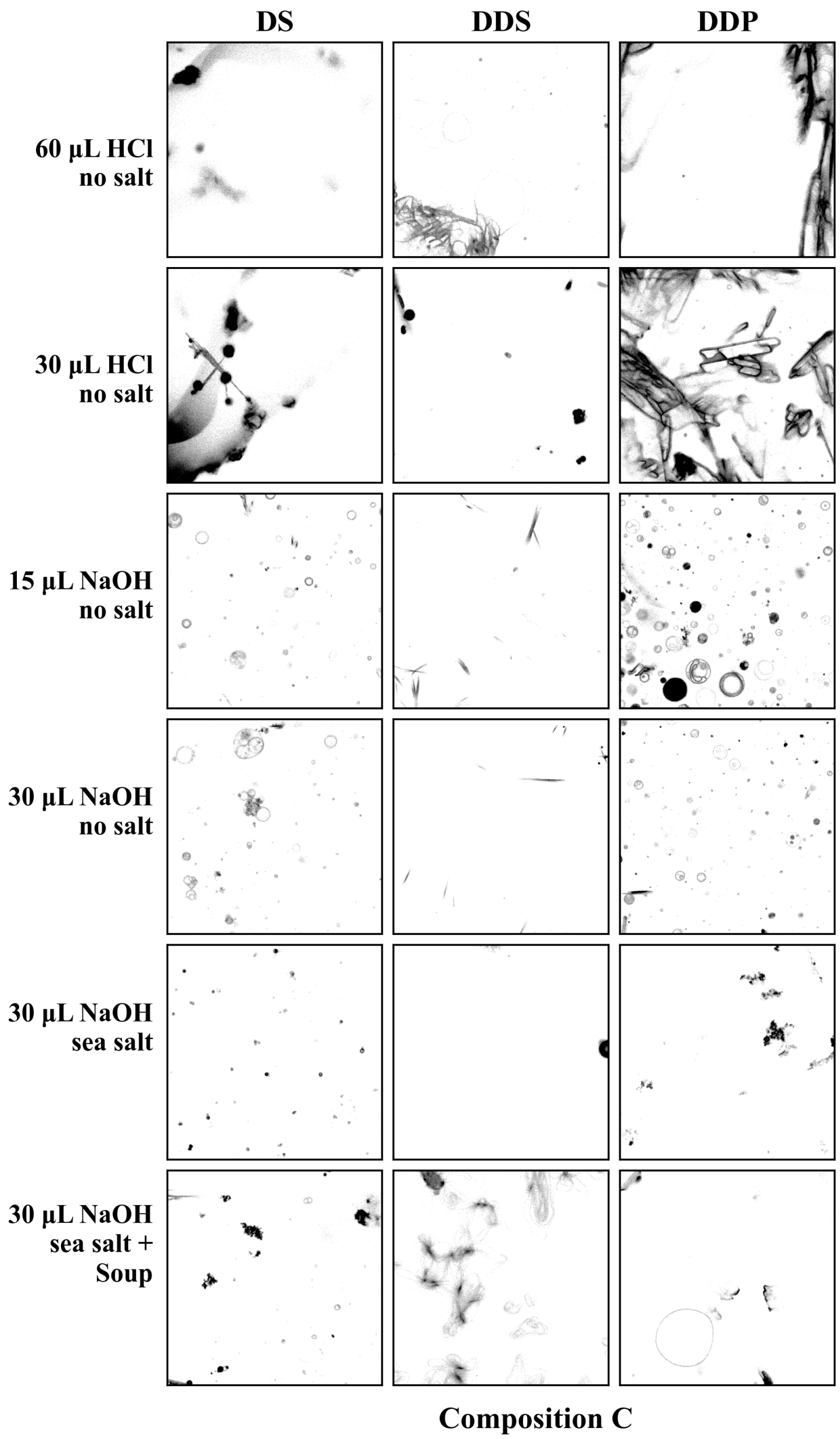

Figure S4: Confocal micrographs of sample morphology with composition C.

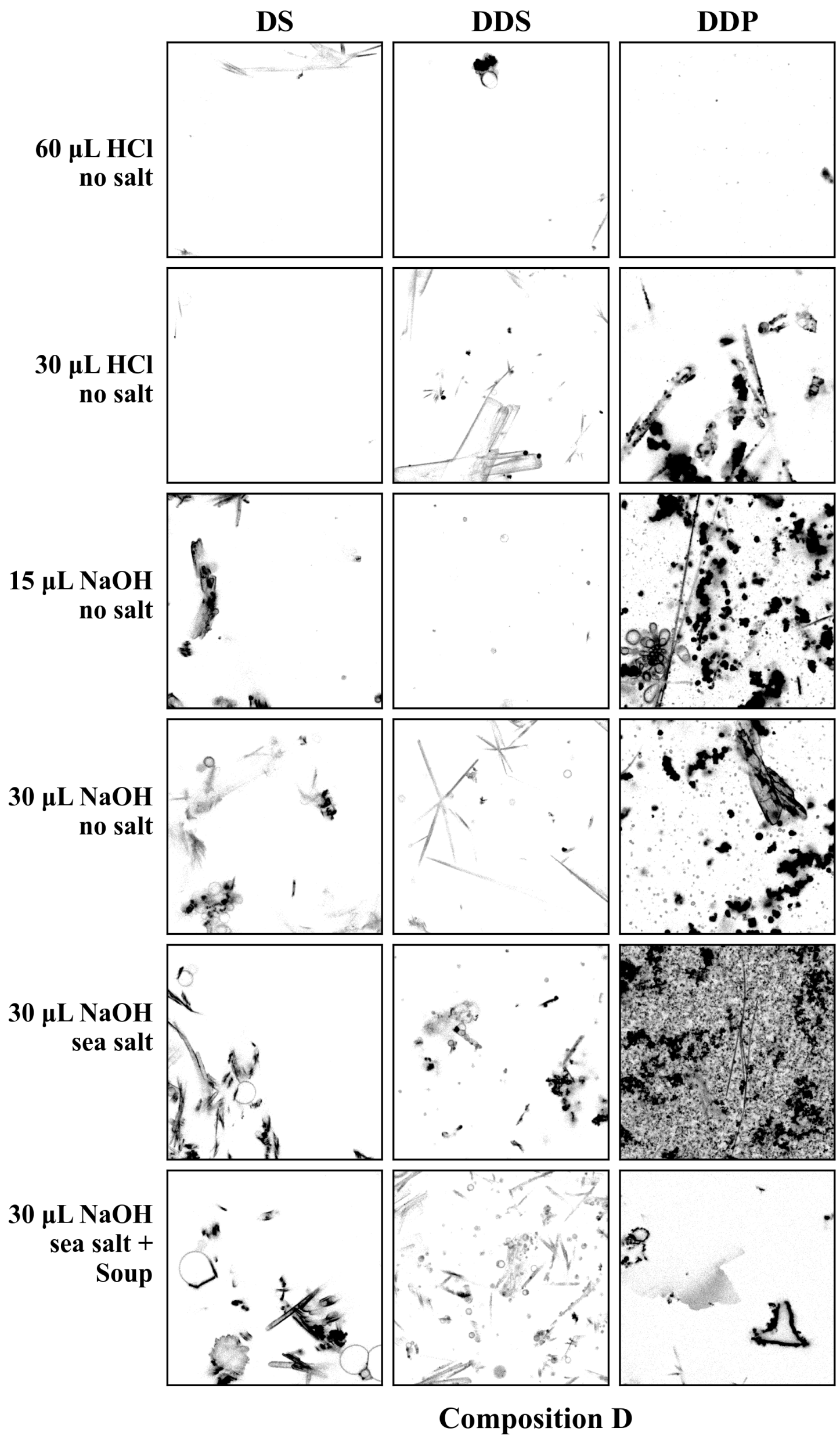

Figure S5: Confocal micrographs of sample morphology with composition D.

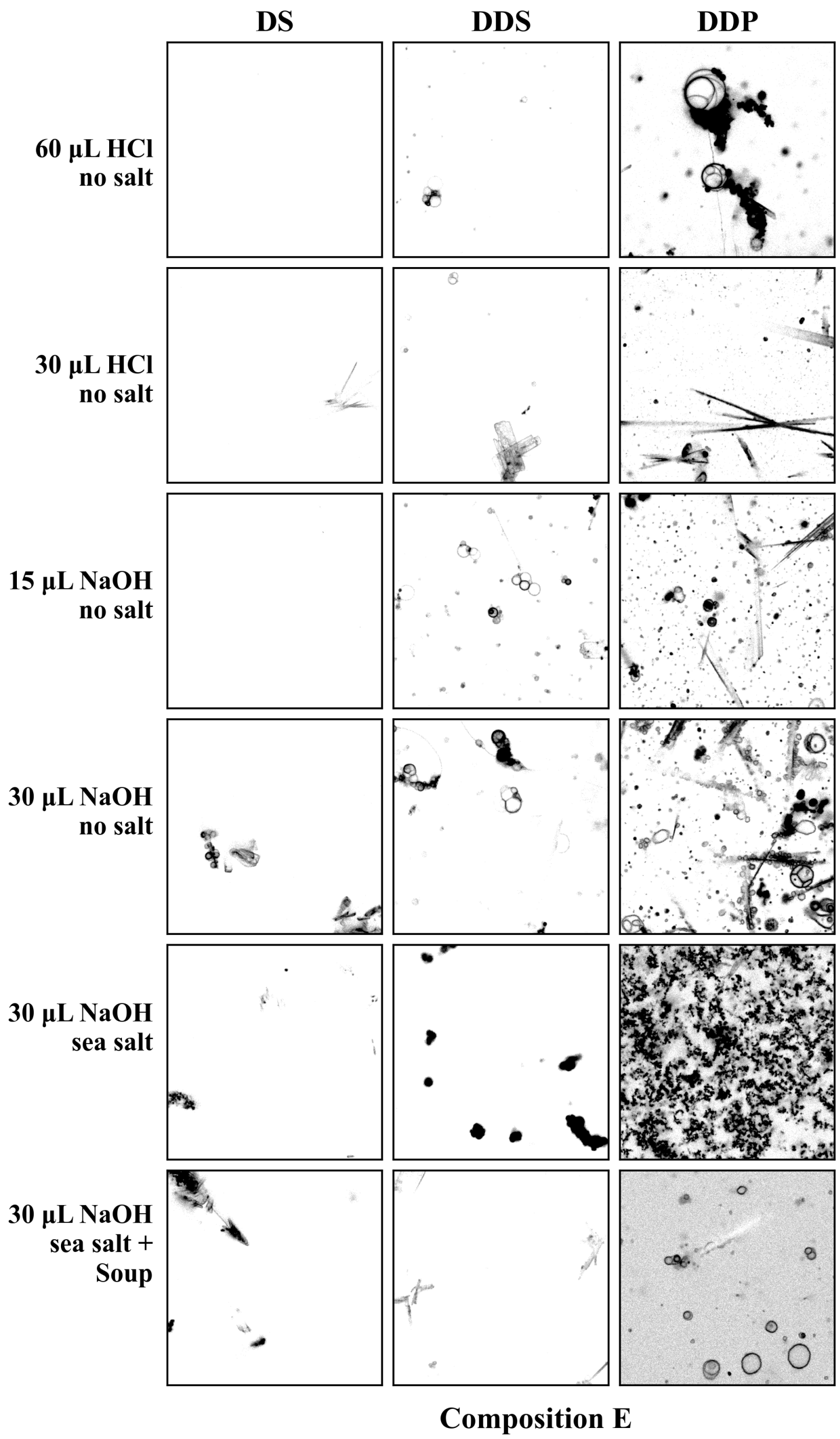

Figure S6: Confocal micrographs of sample morphology with composition E.

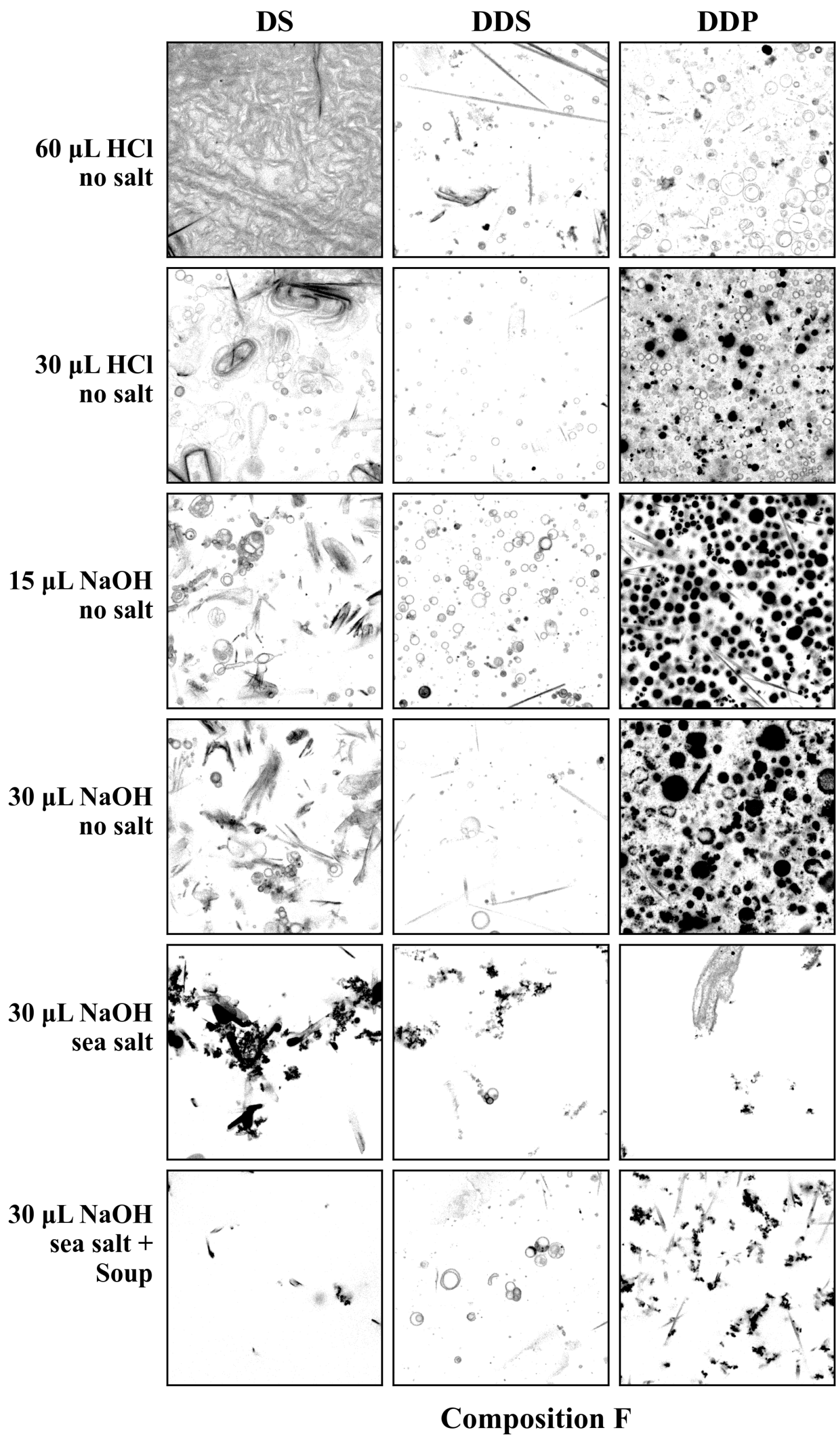

Figure S7: Confocal micrographs of sample morphology with composition F.

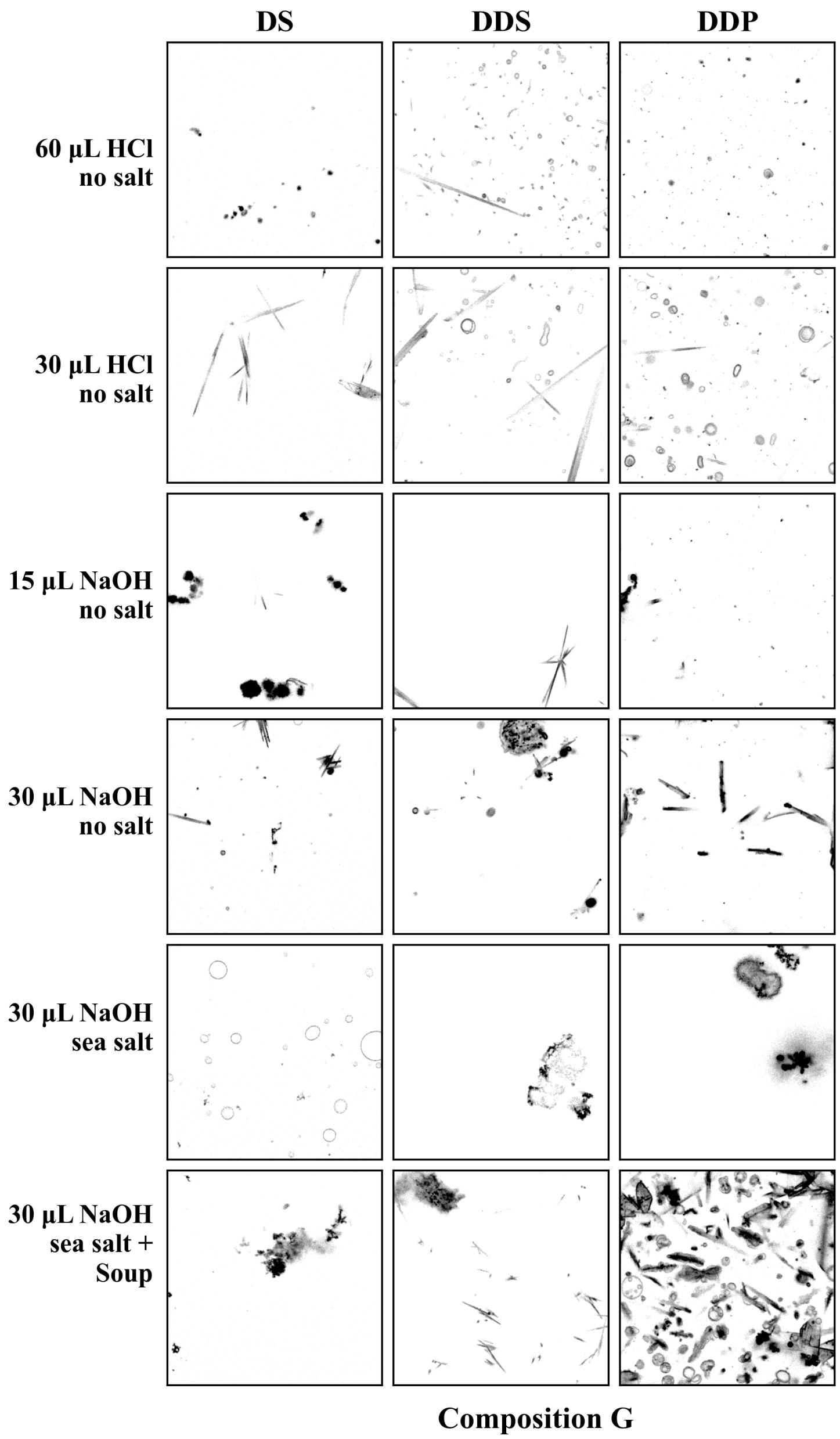

Figure S8: Confocal micrographs of sample morphology with composition G.

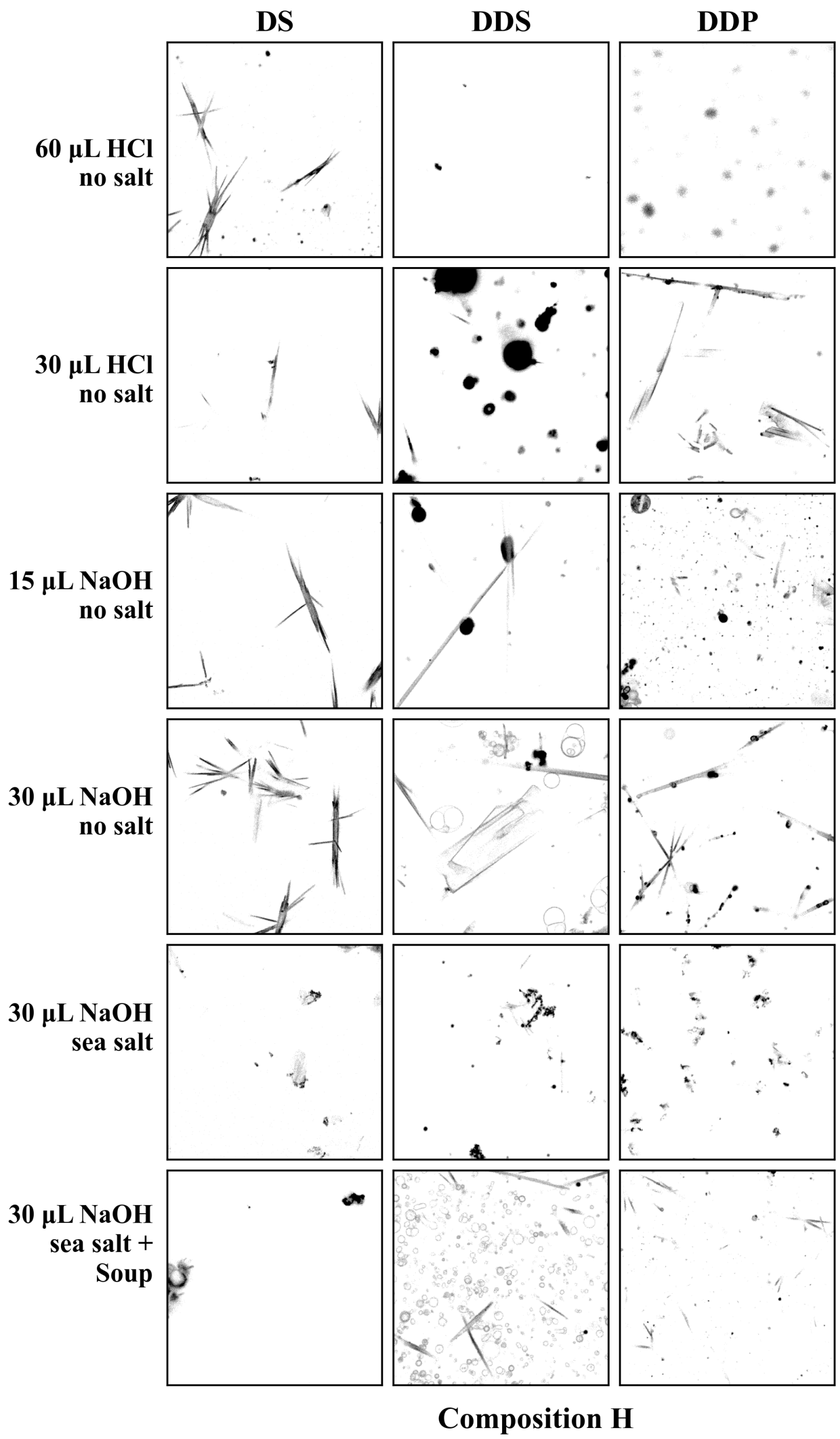

Figure S9: Confocal micrographs of sample morphology with composition H.
